# Structural and Biomolecular Analyses of Borrelia burgdorferi BmpD Reveal a Substrate-Binding Protein of an ABC-Type Nucleoside Transporter Family

**DOI:** 10.1128/IAI.00962-19

**Published:** 2020-03-23

**Authors:** Julia Cuellar, Mia Åstrand, Heli Elovaara, Annukka Pietikäinen, Saija Sirén, Arto Liljeblad, Gabriela Guédez, Tiina A. Salminen, Jukka Hytönen

**Affiliations:** a Institute of Biomedicine, Faculty of Medicine, University of Turku, Turku, Finland; b Turku Doctoral Programme of Molecular Medicine, Faculty of Medicine, University of Turku, Turku, Finland; c Structural Bioinformatics Laboratory, Biochemistry, Faculty of Science and Engineering, Åbo Akademi University, Turku, Finland; d National Doctoral Programme in Informational and Structural Biology, Faculty of Science and Engineering, Åbo Akademi University, Turku, Finland; e Laboratory of Synthetic Drug Chemistry, Institute of Biomedicine, University of Turku, Turku, Finland; f Biochemistry, Faculty of Science and Engineering, Åbo Akademi University, Turku, Finland; g Laboratory Division, Clinical Microbiology, Turku University Hospital, Turku, Finland; Washington State University

**Keywords:** ABC transporter family, BmpD, *Borrelia burgdorferi*, immunization, ligand-binding assay, purine metabolism, substrate-binding protein, X-ray crystallography

## Abstract

Borrelia burgdorferi *sensu lato*, the causative agent of tick-borne Lyme borreliosis (LB), has a limited metabolic capacity and needs to acquire nutrients, such as amino acids, fatty acids, and nucleic acids, from the host environment. Using X-ray crystallography, liquid chromatography-mass spectrometry, microscale thermophoresis, and cellular localization studies, we show that basic membrane protein D (BmpD) is a periplasmic substrate-binding protein of an ABC transporter system binding to purine nucleosides. Nucleosides are essential for bacterial survival in the host organism, and these studies suggest a key role for BmpD in the purine salvage pathway of B. burgdorferi sensu lato. Because B. burgdorferi sensu lato lacks the enzymes required for *de novo* purine synthesis, BmpD may play a vital role in ensuring access to the purines needed to sustain an infection in the host. Furthermore, we show that, although human LB patients develop anti-BmpD antibodies, immunization of mice with BmpD does not confer protection against B. burgdorferi sensu lato infection.

## Abstract (precis)

Borrelia burgdorferi *sensu lato*, the causative agent of tick-borne Lyme borreliosis (LB), has a limited metabolic capacity and needs to acquire nutrients, such as amino acids, fatty acids, and nucleic acids, from the host environment. Using X-ray crystallography, liquid chromatography-mass spectrometry, microscale thermophoresis, and cellular localization studies, we show that basic membrane protein D (BmpD) is a periplasmic substrate-binding protein of an ABC transporter system binding to purine nucleosides.

## INTRODUCTION

Lyme borreliosis (LB) is a tick-borne infectious disease that is prevalent in North American, European, and Asian countries with moderate climates (1–3). Borrelia burgdorferi *sensu lato* (referred to herein as B. burgdorferi) spirochetes belong to the group of LB-causing bacteria, consisting of about 20 different genospecies; Borrelia burgdorferi *sensu stricto*, Borrelia garinii, and Borrelia afzelii are the major human pathogens (1). B. burgdorferi *sensu stricto* is prevalent in North America, whereas B. afzelii and B. garinii are common in Europe and Asia (1, 2). LB is a multisystem and multistage infection in which a skin lesion called erythema migrans is commonly the first sign of a local infection (2). The erythema migrans occurring around the tick bite site results from the host immune response to replicating bacteria in the skin (1). In the disseminated stage of LB, the bacteria have migrated from the initial entry site in the skin to distant organs, such as the central nervous system, the heart, or the joints (2, 3).

B. burgdorferi circulates between its arthropod tick vector and various vertebrate hosts. Although the host environments are different, B. burgdorferi is able to survive despite its limited metabolic capacities, as reviewed by Radolf and colleagues (4). B. burgdorferi lacks the genes encoding components of many biosynthetic pathways (4, 5). The complex genome of B. burgdorferi consists of one linear chromosome (∼1 Mb) and multiple circular and linear plasmids (∼0.6 Mb in total) (5–7). The chromosome carries the main genes essential for maintaining survival and replication in the tick and the vertebrate host but is devoid of genes encoding enzymes for *de novo* synthesis of amino acids, fatty acids, enzyme cofactors, and nucleic acids (5, 8). Using the purine salvage pathway, B. burgdorferi is able to rescue purine bases, nucleosides, and deoxynucleosides from the host environment and to incorporate the nucleotides into bacterial RNA and the deoxynucleotides into DNA after enzymatic conversion (8). In contrast to the genomes of the relapsing fever spirochetes Borrelia hermsii and Borrelia turicatae, the B. burgdorferi genome does not encode a complete set of purine salvage pathway components, as it lacks the genes of the key enzymes ribonucleotide reductase, hypoxanthine phosphoribosyltransferase, adenylosuccinate synthase, and adenylosuccinate lyase (8).

However, several essential transporters and enzymes that are involved in the purine salvage pathway and are critical for B. burgdorferi infectivity in the vertebrate host have been identified. For example, the transporter proteins BBB22 and BBB23 import purine bases (adenine, guanine, and hypoxanthine) from the host environment into the bacteria and are necessary for B. burgdorferi infection in mice (9, 10). Similarly, GuaA (BBB18) and GuaB (BBB17) are essential enzymes for converting purine bases to GMP and dGMP, vital precursors in the synthesis of RNA and DNA (11).

In addition to purine bases, B. burgdorferi rescues (deoxy)nucleosides from the environment. The host-derived (deoxy)nucleoside monophosphates are dephosphorylated to (deoxy)nucleosides by a nucleotidase, and then an energy-driven transporter system (BB0677, BB0678, and BB0679) translocates the nucleosides into the B. burgdorferi cytoplasm (12). The transporter system, containing two permeases (BB0678 and BB0679) and one ATP-binding protein (BB0677), is one of the many ATP-binding cassette (ABC) transporters involved in nutrient transportation in B. burgdorferi (5).

**FIG 1:**
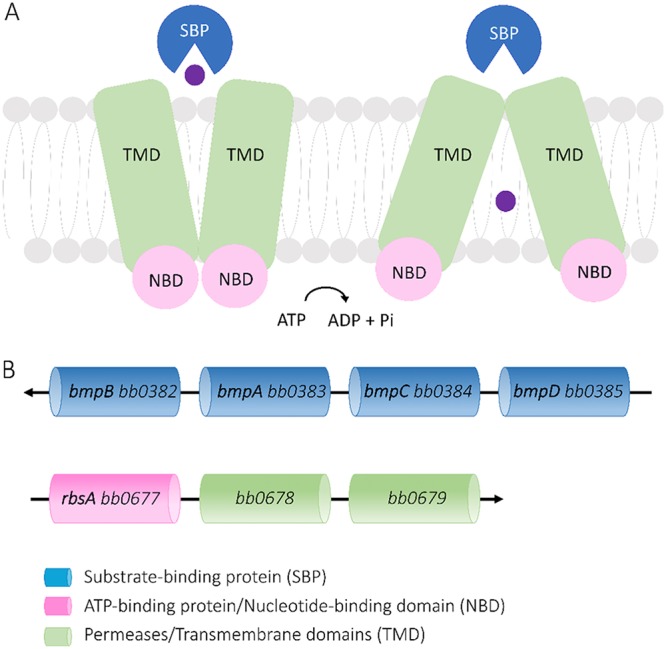
ABC transporter systems, consisting of two TMDs, two NBDs, and an SBP. (A) General function of ABC transporter systems. The SBP, with the substrate molecule (purple), binds to the TMDs, which open in an ATP-dependent manner and allow the passage of the substrate through the cell membrane. (B) Genome organization of the B. burgdorferi ABC transporter components.

The ABC transporters belong to one of the largest families of transporter proteins that use the hydrolysis of ATP to transport various substrates across cell membranes (13). They consist of two transmembrane domains (TMD), forming a translocation channel through the membrane, and two nucleoside-binding domains (NBD), which bind to and hydrolyze ATP (Fig. 1A) (13). In bacteria, ABC transporters play a vital role in the import of nutrients and require a substrate-binding protein (SBP) to deliver the substrate to the translocation channel formed by the two permeases (14). SBPs bind to their substrates with high affinity and specificity and, using the Venus flytrap mechanism (15), they change into a closed conformation when the substrate is bound. This closed conformation is recognized by the TMDs and triggers ATP hydrolysis and opening of the translocation channel (Fig. 1A) (16). Using homology modeling, we recently showed that the four members of a paralogous basic membrane protein (Bmp) family of B. burgdorferi *sensu stricto*, namely, BmpA, BmpB, BmpC, and BmpD (BB0383, BB0382, BB0384, and BB0385, respectively), belong to the SBPs of an ABC transporter family (Fig. 1B) (17). Furthermore, we predicted that BmpA to BmpD are likely involved in the uptake of purine nucleosides, based on their structural similarities to PnrA, a purine nucleoside-binding protein of the related spirochete Treponema pallidum (17, 18).

Efforts to determine the exact function of the Bmp proteins have so far given contradictory results. BmpA to BmpD have been described as laminin-binding proteins expressed on the outer surface of B. burgdorferi (19); however, the same proteins have been annotated as ABC transporters for simple sugars, such as ribose or galactose (20, 21). Furthermore, BmpA and BmpB have been suggested to regulate joint inflammation *in vivo* (22). A comprehensive protein localization study showed that BmpB, BmpC, and BmpD are expressed on the inner membrane in the periplasmic space of B. burgdorferi (23), arguing against an adhesin role for the proteins (24). Because these earlier studies yielded discrepant results, we focused on BmpD as a representative member of the Bmp family, to shed light on the role of these proteins in the physiology of B. burgdorferi. Additionally, thoroughly characterized B. burgdorferi proteins are potential key vaccine candidates for LB prevention.

In this study, we solved the crystal structure of BmpD and analyzed its nucleoside-binding properties. Our results indicate that BmpD functions as an SBP of the ABC-type transporter family, importing purine nucleosides from the environment into the bacterial cell. Furthermore, we show that human LB patients develop antibodies against BmpD but, based on mouse immunization studies, immunity against BmpD does not confer protection from LB.

## RESULTS

### BmpD is an ABC-type SBP.

**FIG 2:**
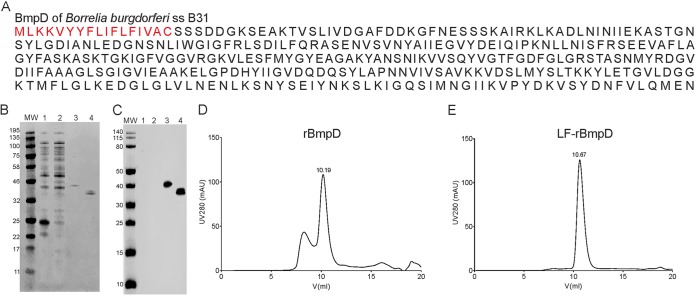
Expression and purification of BmpD. (A) Amino acid sequence of BmpD of Borrelia burgdorferi *sensu stricto* B31. The amino acids marked in red indicate the signal peptide sequence not included in rBmpD. (B and C) Detection of rBmpD by SimplyBlue staining (B) and Western blotting (C). Lanes 1, untransformed E. coli BL21(DE3) pLys cells; lanes 2, cells expressing BmpD without induction; lanes 3, purified rBmpD (39 kDa); lanes 4, purified LF-rBmpD (37 kDa). The molecular weight markers (MW) indicate the protein sizes, in kilodaltons. (D and E) Chromatograms of rBmpD (D) and LF-rBmpD (E) after purification by SEC. The elution volume of the protein of the correct size is indicated above the corresponding peak.

**FIG 3:**
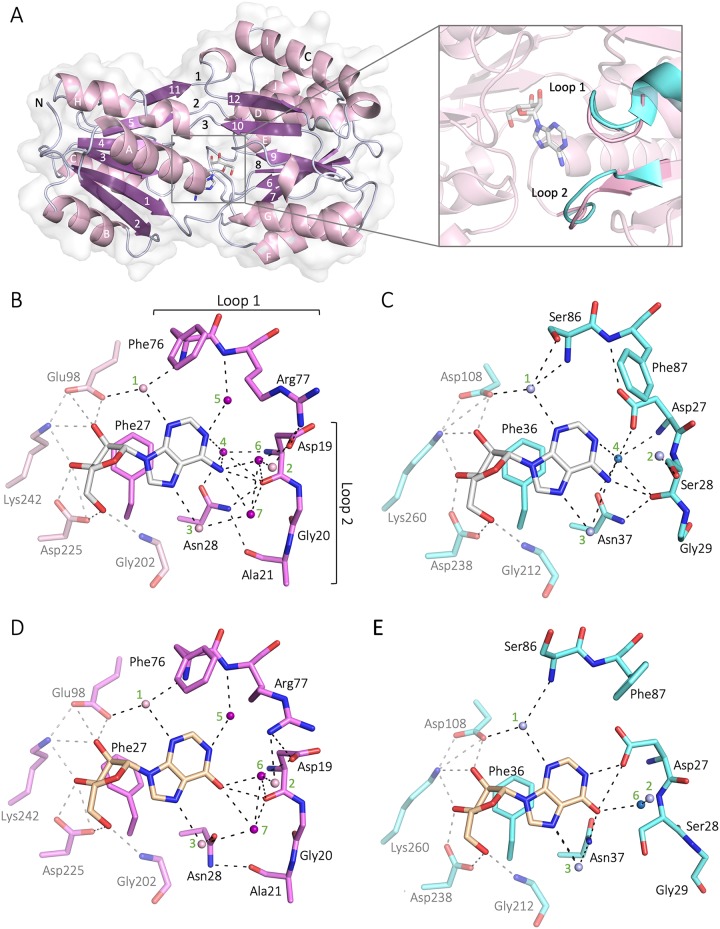
(A) Structure of B. burgdorferi *sensu stricto* B31 BmpD with adenosine solved by X-ray crystallography. The beta sheets in the center of each domain are shown in purple (1 to 12) and the surrounding alpha helices in light pink (A to J). The ligand (adenosine) is shown as white sticks in the cleft between the domains, and the three loops (loops 1 to 3) connecting the two domains are shown above the ligand. The two loops contributing to the binding site differences are shown in the closeup. (B to E) Ligand-binding site comparison of BmpD (B), the PnrA structure with adenosine (PDB accession number 2FQY) (C), the model for the BmpD-inosine complex (D), and the PnrA structure with inosine (PDB accession number 2FQW) (E). All potential hydrogen bonds are shown. The interactions of the base part of the nucleosides are highlighted, since the ribose part forms identical interactions in all of the structures. Adenosine is shown as white sticks and inosine as wheat sticks. Water molecules are shown as spheres (1 to 7); lighter colored spheres are completely conserved, and darker spheres differ between the structures. Phe176 (BmpD) and Phe186 (PnrA) are omitted for clarity.

**FIG 4:**
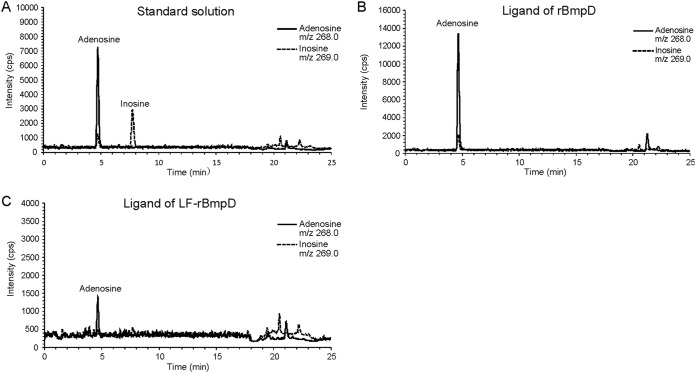
Detection of bound ligand of BmpD by LC-MS. LC-MS chromatograms of the standard solution containing both adenosine and inosine, with retention times of 4.7 and 7.7 min, respectively (A), the adenosine bound to rBmpD under native conditions (B), and the residual adenosine bound to LF-rBmpD after denaturation and refolding (C).

To purify BmpD for ligand-binding and crystallization experiments for X-ray structure determination, we expressed recombinant BmpD (rBmpD) in Escherichia coli without the signal peptide sequence (Fig. 2A) and purified it by affinity chromatography, under native conditions, and size exclusion chromatography (SEC) (Fig. 2D). The calculated size of rBmpD is 39 kDa (Fig. 2B and C, lanes 3). The purified protein was successfully crystallized, and the BmpD structure was solved by molecular replacement. The final crystal structure of BmpD was refined to 1.43 Å (Fig. 3A and Table 1). The structure is a monomer and consists of two domains connected by a linker region, characteristic of the SBPs (25). The N-terminal domain consists of residues 8 to 115 and 243 to 269, and the C-terminal domain consists of residues 116 to 242 and 270 to 323. The BmpD structure is similar to the structures of the other ABC-type SBPs, which bind specific substrate molecules and transfer them to membrane-bound ABC transporters that transport the substrates into the bacteria (26). The SBPs are characterized by two alpha/beta domains containing a central beta sheet surrounded by alpha helices. In BmpD, the central beta sheet of both domains contains six beta strands and the N-terminal domain has four alpha helices, whereas the C-terminal domain has six (Fig. 3A). The crystal structure also unexpectedly contained an endogenously bound ligand, which had not been added during the crystallization setup. The ligand was bound in the cleft between the two domains, and the electron density indicated that the ligand was a purine nucleoside, which was confirmed to be an adenosine by liquid chromatography-mass spectrometry (LC-MS) analysis (Fig. 4A and B).

**TABLE 1:** Data collection and refinement statistics

Parameter	Value(s)^*a*^
Diffraction source	ESRF ID30A-3
Detector	Eiger
Wavelength (Å)	0.9677
Resolution range (Å)	30.7–1.43 (1.481–1.43)
Space group	C 1 2 1
Unit cell	a=106.78 Å, b=42.90 Å, c=66.38 Å, α=90° β=117.34° γ=90°
Total no. of reflections	343,017 (12,968)
No. of unique reflections	44,953 (2,652)
Multiplicity	7.6 (4.9)
Completeness	0.91 (0.51)
Mean *I/*σ*I*	19.35 (3.86)
Wilson *B*-factor	11.44
*R*_meas_	0.06703 (0.3546)
*CC*_1/2_	0.999 (0.915)
No. of reflections used in refinement	44,944 (2,652)
No. of reflections used for *R*_free_	2,193 (154)
*R*_work_	0.1538 (0.1856)
*R*_free_	0.1792 (0.2163)
No. of nonhydrogen atoms	2,889
Protein	2,459
Ligand	19
Ion	2
Water	409
RMSD	
Bond lengths (Å)	0.005
Bond angles (°)	0.80
Ramachandran analysis (%)	
Favored	96.6
Allowed	3.4
Outliers	0
Average *B*-factor	17.28
Protein	14.94
Ligand, ion	10.36
Water	31.76

a Statistics for the highest-resolution shell are shown in parentheses.

Superimposition of the B. burgdorferi *sensu stricto* B31 BmpD and T. pallidum PnrA (PDB accession number 2FQY) (18) structures revealed a root mean square deviation (RMSD) of 1.0 Å, indicating very high structural similarity, although the proteins share amino acid sequence identity of only 27.8% (17). The ligand-binding site is also highly conserved, as described in our previous study (17). Both proteins are bound to a purine nucleoside, which forms hydrogen bonds to surrounding residues and water molecules. Furthermore, the aromatic rings of the purine base form stacking interactions with two phenylalanines. Compared to PnrA, BmpD has a more extensive water-mediated hydrogen-bonding network connecting the purine base of the nucleoside with Asp19 and Asn28 (Fig. 3B and C; also see Fig. S1 in the supplemental material).

The main differences between the adenosine-binding sites in BmpD and PnrA are found in the loops flanking the binding site (Fig. 3A). In loop 1, Ser86 and Phe87 in PnrA (Fig. 3C) are exchanged for Phe76 and Arg77 in BmpD (Fig. 3B). Asp27 in loop 2 of PnrA points toward the ligand and makes a hydrogen bond with the backbone nitrogen of Phe87, whereas Asp19 in BmpD is turned away from the binding site and forms ionic interactions with Arg77. Compared to that of BmpD (Fig. 3A and B), loop 2 of PnrA is longer by 1 residue and thus intrudes more deeply into the binding site (Fig. 3C).

The only difference between adenosine and inosine is the amino and carbonyl groups, respectively, of the base part (Fig. 3C and E). To analyze how BmpD could bind inosine, we thus created a model for inosine-bound BmpD (Fig. 3D), based on the PnrA-inosine complex structure (PDB accession number 2FQW). Comparison of the inosine- and adenosine-bound structures of PnrA showed that inosine binding results in a conformational change in loop 2 (Fig. 3C and E). As a result, the side chain of Ser28 turns away from the binding site and its position is replaced by water molecule 6. In addition, water molecule 4 is excluded, allowing direct interaction between the carbonyl oxygen of inosine and Asp27 in PnrA (Fig. 3E). In the BmpD-inosine model, loop 2 may remain unchanged as Ser28 is replaced by Gly20 in BmpD, and the carbonyl oxygen of inosine makes hydrogen bonds with water molecule 6 and the main-chain oxygen of Asp19 (Fig. 3D).

### BmpD binds to a nucleoside.

**FIG 5:**
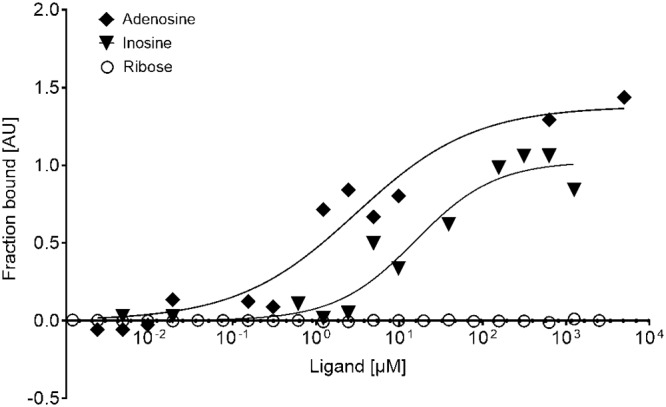
Ligand-binding properties of LF-rBmpD analyzed by label-free MST. The binding curves are representative dose-response curves for one measurement of adenosine and inosine binding to LF-rBmpD. The concentration of LF-rBmpD was constant at 500 nM, while the concentrations of the ligands varied from 1.2 nM to 5 mM. Ribose, the negative control, did not produce a binding curve.

Because the solved crystal structure of BmpD and the LC-MS analysis of the purified protein confirmed that BmpD binds to a purine nucleoside, we further analyzed its nucleoside-binding properties using microscale thermophoresis (MST) analyses. The endogenously bound adenosine was nearly completely removed from rBmpD by denaturation and refolding before SEC purification, as only a residual amount of bound adenosine remained (Fig. 2E and 4C). The size of the refolded ligand-free rBmpD (LF-rBmpD) was smaller than that of the original protein (approximately 37 kDa) (Fig. 2B and C, lanes 4). The protein yield was low after the denaturation treatment. Hence, MST was chosen for the ligand-binding assay because it consumes only small amounts of the protein. LF-rBmpD was mixed with adenosine, inosine, guanosine, xanthosine, or ribose, and the diffusion in a thermal gradient was monitored as a function of ligand concentration (27). The resulting data demonstrated that LF-rBmpD bound with higher affinity to adenosine than to inosine, while no binding to ribose, the negative-control ligand, could be detected (Fig. 5). No MST binding curves could be obtained for LF-rBmpD with guanosine or xanthosine (data not shown).

### BmpD is located in the periplasmic space.

**FIG 6:**
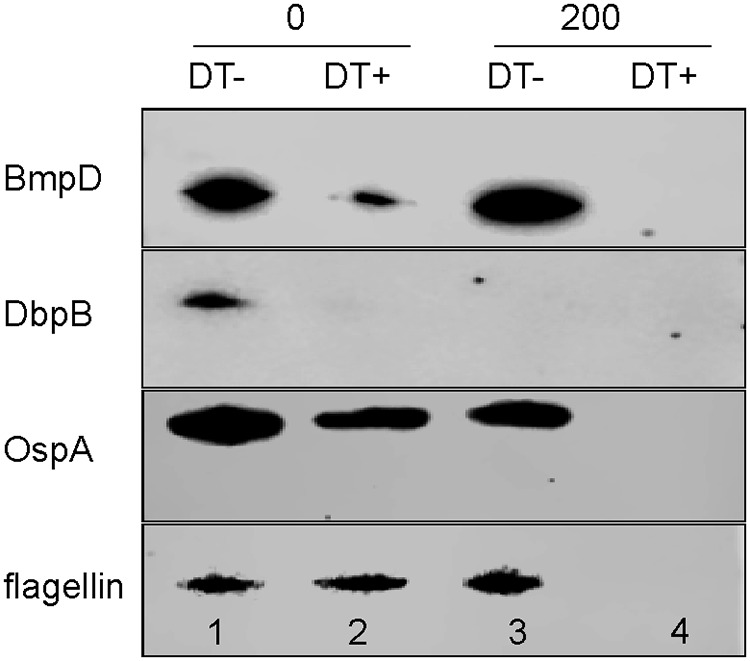
Cellular localization of BmpD in B. burgdorferi *sensu stricto* B31. BmpD, decorin-binding protein B (DbpB), outer surface protein A (OspA), and flagellin were detected by Western blotting after incubation of bacterial cells with proteinase K at concentrations of 0 or 200 μg/ml, in the absence (DT-) or presence (DT+) of the detergent Triton X-100.

SBPs are known to be located in the periplasmic space of Gram-negative bacteria, where they transfer the substrate molecules to membrane-bound transporters for passage into the cytoplasm (26). The amino acid sequence of BmpD includes a signal peptide guiding the export of BmpD outside the bacterial cytoplasm (Fig. 2A) (28). Hence, BmpD is expressed either in the bacterial periplasm or on the outer surface. Furthermore, BmpD was not degraded by proteinase K, whereas the known surface-exposed proteins DbpB and OspA were degraded, as seen by the decreased Western blot signals (Fig. 6, lane 3) (23, 29). In the presence of a detergent and proteinase K, however, BmpD was degraded similarly to the known periplasmic flagellin (Fig. 6, lane 4). Thus, BmpD is likely to be expressed in the periplasmic space.

### BmpD is expressed during human LB, as indicated by anti-BmpD antibodies in patient sera.

**FIG 7:**
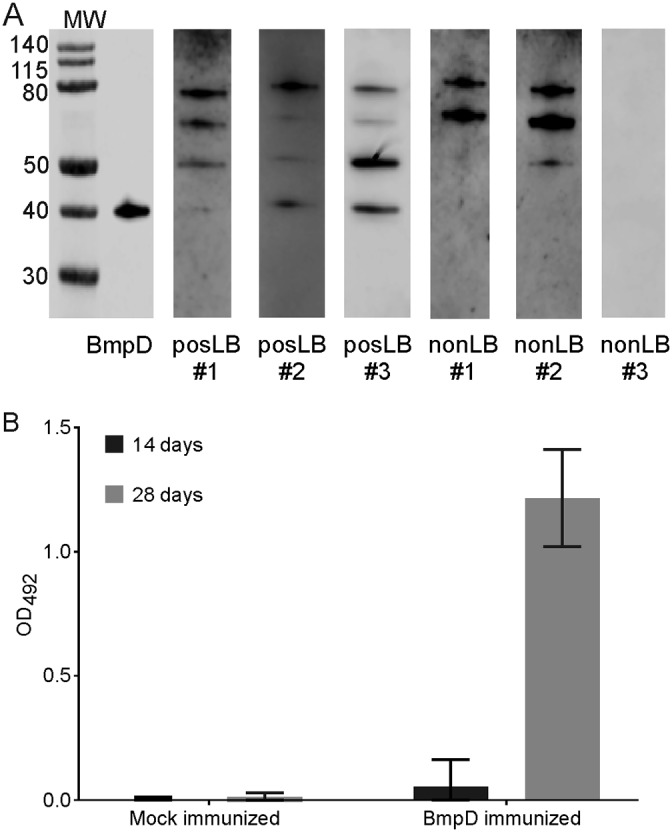
BmpD immunogenicity in human patients and in mice. (A) Antibodies to purified rBmpD (0.5 μg) were detected by Western blotting in serum samples from confirmed LB patients (posLB) (*n* = 3) and non-LB patients (nonLB) (*n* = 3). The correct size of BmpD (39 kDa) is shown with anti-BmpD staining, as a control, in the first blot on the left. The signals in the size range of 50 to 80 kDa originate from E. coli proteins. The molecular weight markers (MW) indicate the protein sizes, in kilodaltons. (B) Levels of IgG to BmpD in mouse serum samples after BmpD immunization, at 14 and 28 days after the first immunization, were measured by ELISA. The data are expressed as OD_492_ values and are presented as bars with the median and range of IgG antibody levels in each study group.

To evaluate the role of BmpD expression in B. burgdorferi survival in the host, sera from randomly selected LB patients were tested for BmpD antibodies. BmpD antibodies were detected in sera from LB patients (Fig. 7A). Although the intensity of the signals for antibodies recognizing rBmpD varied among the samples, these results showed that B. burgdorferi expresses BmpD during human infection, whereas the sera of non-LB patients did not recognize BmpD.

### BmpD is immunogenic in mice, but BmpD immunization does not confer protection from B. burgdorferi infection.

Next, we wanted to study whether BmpD-immunized mice were protected against B. burgdorferi infection. C3H/HeN mice were actively immunized with either rBmpD (BmpD immunized) or adjuvant only (mock immunized), and sera were collected. The BmpD-immunized mice had developed high IgG antibody levels toward rBmpD at 28 days (Fig. 7B). For the passive immunization studies, the sera collected from BmpD- and mock-immunized mice were transferred to a second set of mice before B. burgdorferi *sensu stricto* B31 challenge. In addition, two groups of control mice pretreated with saline either were challenged with B. burgdorferi *sensu stricto* B31 (positive control) or received a phosphate-buffered saline (PBS) injection (negative control).

**FIG 8:**
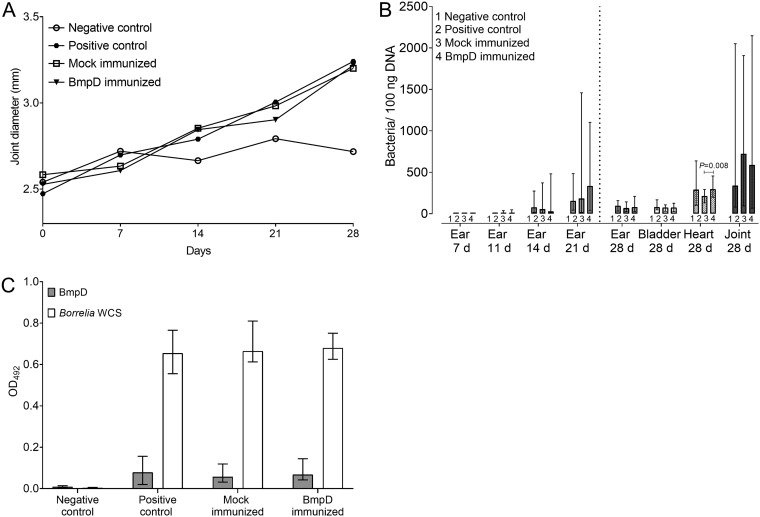
Passive immunization of mice with serum containing BmpD antibodies. (A) Weekly progression of joint swelling in mice in the different study groups. The data are expressed as the mean diameters of the joints of all mice per study group. (B) B. burgdorferi loads in tissue samples from mice in the different study groups, as analyzed by qPCR. The bars to the left of the dotted vertical line indicate results of the ear biopsy samples at different time points (d, days), and those to the right indicate results from tissue samples collected at the end of the study. The data are expressed as the number of bacterial genome copies per 100 ng extracted DNA. The bars indicate the median and range for each study group. The Kruskal-Wallis test and the Steel-Dwass *post hoc* test were used for statistical analyses. *P* values of <0.05 are considered statistically significant. (C) Levels of IgG antibodies to rBmpD and B. burgdorferi WCS in mouse serum samples from different study groups, as measured by ELISA. The data are expressed as OD_492_ values and are presented as bars with the median antibody level and range for each study group. The Kruskal-Wallis test was used for statistical analyses. *P* values of <0.05 are considered statistically significant.

During the study, joint swelling was monitored weekly, because B. burgdorferi *sensu stricto* B31 causes significant arthritis (2). As expected, the mice in the positive-control group developed joint swelling, starting on day 14 and persisting until day 28 (Fig. 8A). The mice in the BmpD- and mock-immunized study groups developed joint swelling similar to that of the positive-control mice. A small increase in joint diameter was observed for the negative-control mice, due to the growth of the mice.

The B. burgdorferi infection status of the mice was further analyzed by culturing and quantifying the bacterial loads in the mouse tissue samples and by performing serological analyses. Starting from day 21 and lasting until the end of the study, all mice in the BmpD- and mock-immunized and positive-control groups were culture positive (Table 2). There were no statistically significant differences in the bacterial loads in ear tissue samples from the three B. burgdorferi *sensu stricto* B31-challenged study groups at days 7, 11, 14, and 21 and in the ear, bladder, and joint tissue samples collected at day 28 (*P* = 0.243, *P* = 0.589, *P* = 0.506, *P* = 0.730, *P* = 0.182, *P* = 0.571, and *P* = 0.218, respectively) (Fig. 8B). Although the difference was small, the bacterial load in the heart tissue was statistically significantly greater in the BmpD-immunized mice than in the mock-immunized mice (*P* = 0.008) (Fig. 8B). Also, all mice in the three B. burgdorferi *sensu stricto* B31-challenged study groups developed IgG antibodies toward B. burgdorferi whole-cell sonicate (WCS) (*P* = 0.467) and toward rBmpD (*P* = 0.324), without statistically significant differences among the three groups (Fig. 8C). The negative-control mice remained uninfected, as all tissue samples were negative by B. burgdorferi culture and quantitative PCR (qPCR) (Fig. 8B and Table 2) and no IgG antibodies toward B. burgdorferi WCS or rBmpD could be detected (Fig. 8C).

**TABLE 2:** Number of positive B. burgdorferi cultures among all studied tissue samples from mice, according to study group

Study group	No. of positive cultures/total no. of samples								
	Postinfection				At study end				
	Day 7	Day 11	Day 14	Day 21	Ear	Heart	Bladder	Joint	Any tissue
Negative control (*n* = 4)	0/4	0/4	0/4	0/4	0/4	0/4	0/4	0/4	0/4
Positive control (*n* = 10)	0/10	6/10	8/10	10/10	9/10	8/10	10/10	10/10	10/10
Mock immunized (*n* = 12)	0/12	6/12	12/12	12/12	11/12	12/12	12/12	12/12	12/12
BmpD immunized (*n* = 12)	0/12	6/12	9/12	12/12	12/12	12/12	12/12	12/12	12/12

In summary, all BmpD-immunized mice were infected by B. burgdorferi *sensu stricto* B31, as evidenced by culture, detectable B. burgdorferi loads in various tissues, high antibody levels toward B. burgdorferi WCS, and development of joint swelling. Thus, BmpD immunization does not confer protection from B. burgdorferi infection.

## DISCUSSION

Survival and proliferation of infectious bacteria require access to host nutrients, as many pathogens have limited biosynthetic capacity and therefore must use salvage pathways to obtain the nutrients needed (12). B. burgdorferi lacks the essential enzymes for *de novo* synthesis of nucleic acids and acquires purines through the purine salvage pathway (8). Here, we show for the first time that BmpD is a component of a purine nucleoside transporter system of B. burgdorferi. Furthermore, we report that BmpD is expressed during B. burgdorferi infection in humans. However, antibodies toward BmpD do not confer protection against B. burgdorferi infection.

B. burgdorferi has a fragmented and rather small genome composed of one linear chromosome and multiple plasmids, which can be lost during long-term *in vitro* culturing (5). The linear chromosome carries genes essential for bacterial metabolism and replication (5, 30). The plasmids contain genes encoding mainly virulence factors and are not required for bacterial growth *in vitro* except for circular plasmid 26, which carries the *guaAB* operon (9). B. burgdorferi is an auxotrophic bacterium that is unable to synthesize *de novo* amino acids, fatty acids, vitamin cofactors, and nucleotides (5, 20). Therefore, B. burgdorferi survival depends on the transportation of vital molecules from the host environment.

Previously, BmpD was suggested to be important for B. burgdorferi infection in the mammalian host, but with an unspecified function (19, 24, 31). The chromosomal localization of the *bmpD* gene, the conserved amino acid sequence of BmpD within the B. burgdorferi genospecies (17), and the expression of BmpD during infection in humans suggest that BmpD is essential for B. burgdorferi survival. In this study, we present the crystal structure of BmpD at a resolution of 1.43 Å and describe the role of BmpD as a nucleoside-binding protein involved in the purine salvage pathway. The expression of BmpD in the periplasmic space of B. burgdorferi supports the notion that BmpD functions as an SBP.

**FIG 9:**
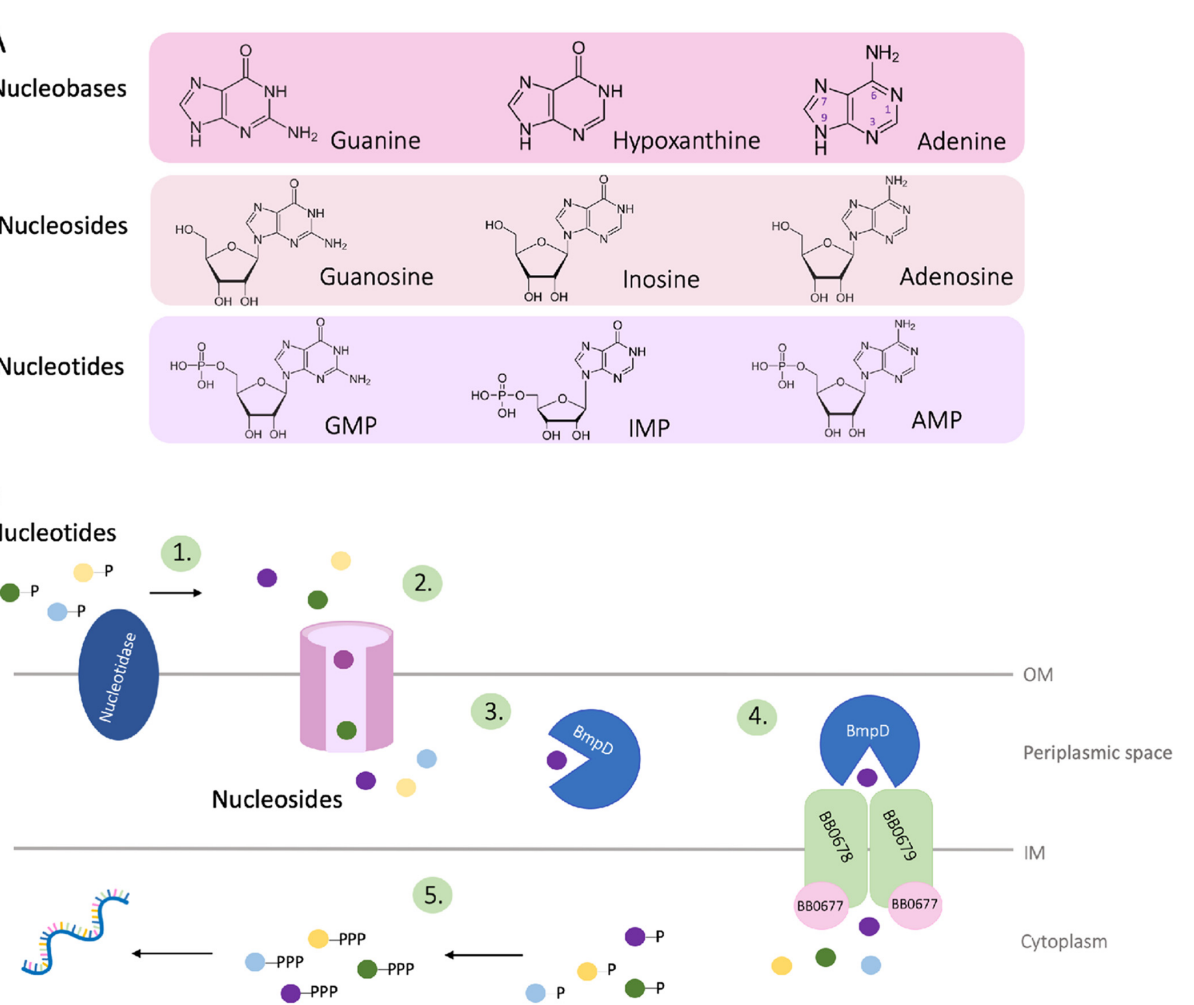
(A) Two-dimensional structures of nucleobases, nucleosides, and nucleotides (nucleoside monophosphates). The atom-numbering convention of nucleobases is shown in the adenine structure. (B) Schematic view of the purine salvage pathway in B. burgdorferi. Step 1, a nucleotidase converts nucleotides (i.e., GMP, IMP, and AMP) into nucleosides (guanosine, inosine, and adenosine) by removing the phosphate. Steps 2 and 3, the nucleosides are then transported through the outer membrane into the periplasmic space, where they bind to SBPs, like BmpD. Step 4, the SBP transports the nucleoside to a membrane-bound ABC transporter system (BB0677 to BB0679), where it is transported into the cytoplasm. Step 5, inside the cytoplasm, deoxynucleotide kinase (BB0239) adds a phosphate to the nucleoside, reforming a nucleoside monophosphate. Adenylate kinase (BB0417) and nucleoside diphosphate kinase (BB0463) then add additional phosphates, forming first nucleoside diphosphates and finally nucleoside triphosphates (ATP and GTP), which are incorporated into RNA.

The crystal structure of BmpD revealed an endogenously bound ligand composed of a purine base and a ribose moiety, resembling a nucleoside. The nucleoside was identified as adenosine by LC-MS analysis. The ligand-binding assay subsequently demonstrated that BmpD also binds to inosine. Despite the differences in the nucleoside structures (Fig. 9A), similar interactions could be formed with adenosine and inosine (Fig. 3B and D). Based on our structural analysis, conformational changes and additional water molecules compensate for differences in the nucleoside structures and ensure that corresponding interactions are formed (Fig. 3). Binding to a group of structurally similar substrates is a common feature among other SBPs (16).

During B. burgdorferi infection in humans, the bacteria salvage nucleobases and nucleosides from the host environment. The physiological concentrations of nucleosides in humans are 0.4 to 6 μM, except for inosine, whose concentration is about 160 μM (32). The higher concentration of inosine reflects its pivotal role in the purine salvage pathway as a precursor of IMP. IMP functions as a central branch point and can be converted into both AMP and GMP and their deoxygenated forms, which are ultimately utilized for the biosynthesis of RNA and DNA, respectively. In contrast to the purine salvage pathway in humans, the purine salvage pathway in B. burgdorferi is distinct, as the necessary enzymes converting IMP to AMP are missing (8). However, B. burgdorferi might compensate for the lack of adenylosuccinate synthase and adenylosuccinate lyase through the uptake of adenosine via BmpD, leading to the formation of AMP, ADP, and finally ATP for RNA incorporation. Similarly, the uptake of inosine via BmpD leads to the formation of GMP, GDP, and GTP for RNA incorporation.

In Fig. 9B, we have visualized the proposed role of BmpD in the B. burgdorferi purine salvage pathway. The host-derived purine nucleoside monophosphates are converted to nucleosides by a nucleotidase (12) before the nucleosides enter the periplasmic space via outer membrane porins, such as p66, allowing the diffusion of hydrophilic small molecules from the environment into the bacterial periplasmic space (33). Thereafter, BmpD, anchored by a fatty acid chain to the inner membrane of B. burgdorferi (23), binds a free purine nucleoside and transports it to the ABC transporter (BB0677 to BB0679). The ABC transporter imports the nucleoside into the bacterial cytoplasm, where it is converted back to a nucleoside monophosphate by deoxynucleotide kinase (BB0239) (20). Adenylate kinase (BB0417) and nucleoside diphosphate kinase (BB0463) add additional phosphates, forming first nucleoside diphosphates and finally nucleoside triphosphates, which can be incorporated into RNA (11).

Previously, it has been shown that BmpD is expressed during B. burgdorferi infection in mice (34). Here, we show that BmpD is also expressed during B. burgdorferi infection in humans, as LB patients developed antibodies to BmpD. Thus, we investigated whether BmpD immunization would protect mice from B. burgdorferi infection. We chose a passive immunization protocol (35, 36), since young mice (4 to 5 weeks of age) are more susceptible to B. burgdorferi infection than older mice (37) and therefore are usually used in B. burgdorferi mouse infection studies. Based on the immunization experiment results, we conclude that antibodies to the periplasmic BmpD do not confer immunity that would protect mice from B. burgdorferi infection. Also, immunization with BmpA or BmpD did not confer protection against B. burgdorferi infection (22).

In conclusion, BmpD is a nucleoside-binding protein of an ABC transporter family that plays a role in the purine salvage pathway of B. burgdorferi. Located in the periplasmic space, BmpD enables B. burgdorferi to acquire purine nucleosides from the host environment. The importance of BmpD as a nucleoside-binding protein for B. burgdorferi survival and for infectivity *in vivo* remains to be determined.

## MATERIALS AND METHODS

### Bacterial strains and growth conditions.

Escherichia coli strains DH5α and BL21 (DE3)pLys (Novagen, Darmstadt, Germany) were cultured at 37°C in Luria-Bertani medium under appropriate antibiotic selection with kanamycin (25 μg/ml; Sigma-Aldrich, Darmstadt, Germany) or chloramphenicol (34 μg/ml; USB Corp., Cleveland, OH, USA). Borrelia burgdorferi *sensu stricto* B31 (a gift from Sven Bergström, University of Umeå, Umeå, Sweden) was cultured at 33°C in Barbour-Stoenner-Kelly II medium.

### Cloning of the *bmpD* gene.

A synthetic *bmpD* gene based on the sequence of B. burgdorferi *sensu stricto* B31 (gene identification number 1195222; residues 18 to 323) was generated by a commercial vendor (Integrated DNA Technologies, Leuven, Belgium). The *bmpD* gene was designed not to include the signal peptide (residues 1 to 17), and the codons were optimized for E. coli. The *bmpD* gene was cloned in the pET-30a(+) vector (Novagen), resulting in a fusion construct with a hexahistidine tag at the N terminus of the recombinant protein. The fusion construct was verified by sequencing. The plasmid was transformed into E. coli DH5α for plasmid amplification and subsequently into E. coli BL21 (DE3)pLys for protein expression.

### Expression and purification of rBmpD.

The E. coli BL21 (DE3)pLys cells were grown until the cell density reached an optical density at 600 nm (OD_600_) of 0.6. Then, protein expression was induced for 3 to 4 h with 1 mM isopropyl-β-d-1-thiogalactopyranoside (IPTG). Cells were harvested by centrifugation and lysed by ultrasonic sonication. The resulting suspension was centrifuged at 8,300 rpm for 30 min at 4°C to remove cell debris. The rBmpD was isolated from the supernatant by affinity chromatography using nickel-nitrilotriacetic acid (Ni-NTA)-agarose (Qiagen, Hilden, Germany), under native conditions.

For crystallization, rBmpD was further purified by SEC with an ÄKTA pure chromatography system (GE Healthcare Life Sciences, Chicago, IL, USA) and a Superdex 75 10/300 GL column (GE Healthcare Life Sciences) equilibrated with 50 mM Tris-HCl (pH 8.0). The peak fractions were analyzed by SDS-PAGE as described below. The fractions containing purified rBmpD were pooled and concentrated with Amicon ultracentrifugal filters (molecular weight cutoff, 10 kDa; EMD Millipore, Burlington, MA, USA), and the protein concentration was determined spectrophotometrically as 9.6 mg/ml. The hexahistidine tag was not removed prior to crystallization.

### Crystallization and X-ray diffraction data collection.

The crystals of rBmpD were obtained by the sitting-drop vapor-diffusion method. After 5 days, the crystals were observed in a 2:1 reservoir solution of 0.2 M sodium chloride, 0.1 M Tris, 20% (wt/vol) polyethylene glycol 6000 (pH 8.0), supplemented with 15% 2-methyl-2,4-pentanediol (MPD) as cryoprotectant. The crystals diffracted to 1.43-Å resolution at beamline ID30A-3 (European Synchrotron Radiation Facility [ESRF], Grenoble, France). Data sets were collected and processed with XDS (38).

### Structure determination and refinement.

The structure of BmpD was solved by molecular replacement with Phaser (39) using T. pallidum PnrA (PDB accession number 2FQY) (18) as the search model, without a ligand. The model-building of the amino acid residues corresponding to BmpD was performed in Coot (40), and the automated refinement cycles were carried out using *phenix.refine* (41). Additional electron density for adenosine, as validated by LC-MS analysis, was observed in the substrate-binding cleft in the initial refined model. The adenosine coordinates of the 2FQY structure (18) were added to the model and included in the next refinement steps. The refinement statistics for the final refined model are listed in Table 1.

### Preparation of LF-BmpD.

To remove the endogenously bound ligand, rBmpD was denatured and refolded (18). E. coli BL21 (DE3)pLys cells expressing rBmpD were lysed and centrifuged, and the supernatant containing rBmpD was allowed to adhere to Ni-NTA-agarose as described above. Then, the Ni-NTA-bound protein was denatured with 10 ml of buffer A (8 M urea, 100 mM Tris-HCl [pH 8.5]) for 1 h at room temperature, washed with 20 ml of buffer A, with 20 ml of buffer A diluted 1:1 and 1:3 with buffer B (20 mM Tris-HCl, 20 mM NaCl, 20 mM imidazole [pH 8.5]), and with 20 ml of buffer B, and finally refolded by incubation with 10 ml of buffer B for 1 h at room temperature. The refolded protein was eluted with 5 ml of buffer C (20 mM Tris-HCl, 20 mM NaCl, 200 mM imidazole [pH 8.5]), concentrated, and purified by SEC as described above. The refolded protein was designated LF-rBmpD.

### LC-MS analysis.

The rBmpD and LF-rBmpD samples were heated and centrifuged, and the supernatant was directly used for LC-MS analysis with an Agilent 1100 series LC system. The analytical method was modified from the method described by Ren and colleagues (42). Separations were conducted using gradient elution on a SunFire C_18_ analytical column (2.1 by 150 mm; particle size, 3.5 μm; Waters, Milford, MA, USA). Mobile phases were 0.1% formic acid in water (solvent A) and 0.1% formic acid in methanol (solvent B). The gradient conditions were 5% solvent B (0 to 12 min), from 5% to 80% solvent B (12 to 13 min), 80% solvent B (13 to 18 min), from 80% to 5% solvent B (18 to 18.5 min), and 5% solvent B (18.5 to 25 min). The flow rate was 0.25 ml/min. Retention times for adenosine and inosine were 4.7 and 7.7 min, respectively (Fig. 4A).

MS detection was performed in selected-ion monitoring mode with a single quadrupole mass spectrometer (HP 1100 LC/MSD). Ionization was based on electrospray ionization in positive-ion mode. The capillary voltage was 4.0 kV, and the drying gas temperature was 350°C. The selected ions for adenosine and inosine were *m/z* 268.0 and 269.0, respectively (Fig. 4A). These masses correspond to the protonated molecules, [M+H]^+^. Adenosine was also detected as *m/z* 269.0, due to its isotopic distribution.

### Microscale thermophoresis.

The binding of nucleosides to LF-rBmpD was monitored with MST (43). Adenosine, guanosine, inosine, xanthosine (Sigma-Aldrich, Darmstadt, Germany), and ribose (negative-control ligand; Sigma-Aldrich) were mixed with LF-rBmpD (final concentration, 500 nM) in a 24-point serial dilution. The concentration of the ligands ranged from 5 mM to 1.2 nM. Samples were filled into zero-background standard-treated capillaries (product number MO-AZ002; NanoTemper Technologies, Munich, Germany) and were measured with Monolith.NT115 LabelFree equipment (NanoTemper Technologies), using 60% light-emitting diode (LED) power and medium MST power. The data were analyzed by MO.Affinity Analysis software (NanoTemper Technologies) and GraphPad Prism (version 8.0; GraphPad Software, San Diego, CA, USA). No dissociation constants are displayed because results of only one experiment are shown.

### Proteinase K assay.

B. burgdorferi *sensu stricto* B31 in mid-logarithmic stage was washed with PBS containing 5 mM MgCl_2_ and diluted to 2 × 10^8^ bacteria/ml. In a total volume of 1 ml, 500 μl of bacterial suspension was incubated for 1 h at room temperature with 0 or 200 μg/ml proteinase K (Sigma-Aldrich), in the absence or presence of detergent (0.05% Triton X-100; Sigma-Aldrich). The bacteria were washed with the aforementioned buffer before analysis of the bacterial lysate samples by Western blotting, as described below.

### SDS-PAGE and Western blotting.

The BmpD protein (0.5 μg) and bacterial lysate samples were electrophoresed though a 10% Bis-Tris polyacrylamide gel (NuPage; Life Technologies, Carlsbad, CA, USA) with morpholineethanesulfonic acid (MES) running buffer containing SDS (Life Technologies). The gels were either stained with SimplyBlue (Invitrogen, Carlsbad, CA, USA) or blotted onto a nitrocellulose membrane. The membrane was incubated for 1 h at room temperature with polyclonal anti-BmpD serum (1:1,000; custom made by Harlan Laboratories, Leicester, UK), polyclonal anti-DbpB serum (1:1,000; custom made by MedProbe, Oslo, Norway), polyclonal p41 antibody (1:1,000; Aviva Systems Biology, San Diego, CA, USA), monoclonal OspA antibody (1:2,500, number H5332; a gift from Sven Bergström, University of Umeå), or human serum samples (1:100) identified as B. burgdorferi antibody positive (*n* = 3) or negative (*n* = 3), using a two-tier testing approach (44). After washing, the membrane was incubated for 1 h at room temperature with horseradish peroxidase (HRP)-conjugated goat anti-rabbit or anti-mouse IgG (1:5,000 or 1:2,000; Santa Cruz Biotechnology, Santa Cruz, CA, USA) or HRP-conjugated rabbit anti-human IgG (1:1,000; Dako Agilent Technologies, Santa Clara, CA, USA). The bound antibodies were detected with WesternBright enhanced chemiluminescence (ECL) HRP substrate (Advansta, San Jose, CA, USA) and an Odyssey Fc imaging system (LI-COR Biotechnology, Bad Homburg, Germany).

### Immunization of mice with BmpD and B. burgdorferi *sensu stricto* infection in immunized mice.

All animal studies were approved by the National Animal Experiment Board in Finland (permission number ESAVI/5507/04.10.07/2014) and were performed in accordance with relevant guidelines and regulations. Four-week-old female C3H/HeN mice (Envigo, Horst, The Netherlands) (*n* = 14) were immunized subcutaneously with 50 μg BmpD with TiterMax Gold adjuvant (Sigma-Aldrich). The control mice (*n* = 15) were mock immunized with adjuvant only. Mice received one booster dose at day 21. Serum samples were obtained from tail veins at day 14 and by cardiac puncture at day 28.

For passive immunization studies, 4-week-old female C3H/HeN mice (Envigo) were intravenously injected with 5 ml/kg mouse serum containing anti-BmpD antibodies, 5 ml/kg serum from mock-immunized mice, or 50 μl saline as a control pretreatment. After 48 h, the BmpD-immunized mice (*n* = 12), mock-immunized mice (*n* = 12), and positive-control mice (*n* = 10) were infected with 10^5^ B. burgdorferi *sensu stricto* B31. The negative-control mice (*n* = 4) received 100 μl PBS. The course of infection was followed by collecting ear biopsy samples at days 7, 11, 14, and 21 postinfection and measuring the lateral diameter of the hind joints, in a blinded manner, once a week. After 28 days postinfection, ear, bladder, heart, and joint samples were collected for *Borrelia* culture and qPCR analyses, as described earlier (45, 46), as well as serum for serological analyses.

### rBmpD and B. burgdorferi WCS serology of mouse samples.

IgG antibodies to rBmpD and B. burgdorferi WCS in the mouse serum samples were measured by enzyme-linked immunoabsorbent assay (ELISA), as described previously (45). Briefly, wells were coated with 10 μg/ml purified rBmpD or 20 μg/ml B. burgdorferi WCS. After serum sample (1:100) incubation, bound IgG was detected with HRP-conjugated goat anti-mouse IgG (1:8,000; Santa Cruz Biotechnologies) and *ortho*-phenylenediamine (OPD) substrate (Kem-En-Tec Diagnostics A/S, Taastrup, Denmark). The reaction was stopped with 0.5 M H_2_SO_4_. The results are expressed as OD_492_ values, and the samples were measured in duplicate.

### Statistical analyses.

The qPCR and serology data, with continuous variables with nonnormality, were analyzed with the Kruskal-Wallis test. Data are presented as bars representing the medians, with ranges indicating the minimum and maximum of each study group. *P* values for the comparisons were corrected using the Steel-Dwass method for multiple comparisons. *P* values of <0.05 were considered statistically significant. Statistical analyses were performed using JMP Pro (version 13.11; SAS Institute Inc., Cary, NC, USA).

### Homology modeling of BmpD bound to inosine.

Inosine was modeled in the BmpD structure based on the inosine-PnrA complex structure (PDB accession number 2FQW) (18).

### Data availability.

The coordinates for the crystal structure of B. burgdorferi *sensu stricto* B31 BmpD have been deposited in PDB (http://www.rcsb.org) with accession number 6SHU.

## Supplementary Material

Supplemental file 1
