# Supplementary material for: Structural and Biomolecular Analyses of Borrelia burgdorferi BmpD Reveal a Substrate-Binding Protein of an ABC-Type Nucleoside Transporter Family
Source: Infect Immun. 2020 Mar 23;88(4):e00962-19. doi: 10.1128/IAI.00962-19 (PMC7093131; doi:10.1128/IAI.00962-19)
Supplement: Supplemental file 1 [file IAI.00962-19-s0001.pdf]

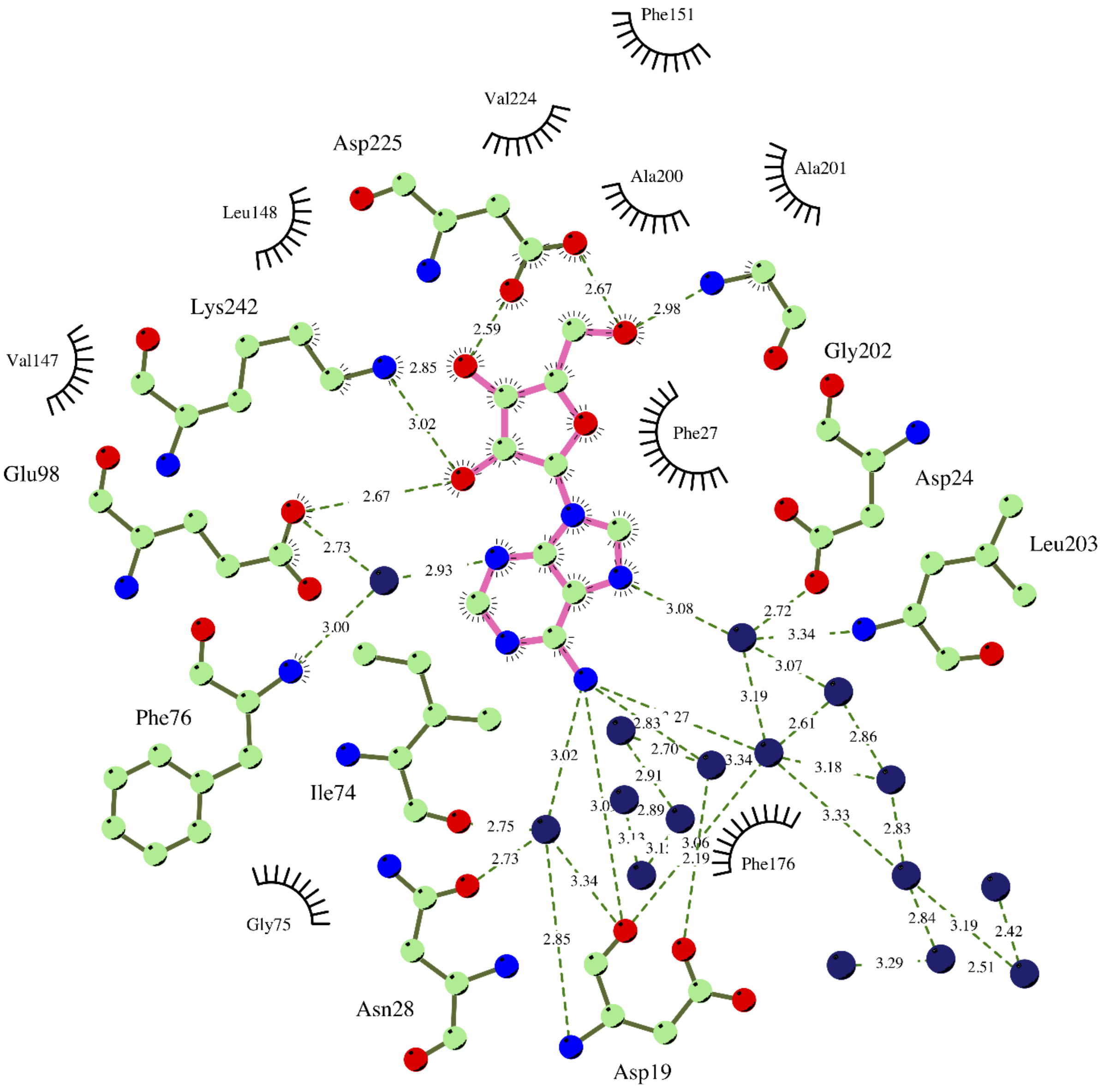

Figure S1. Schematic diagram made by LigPlot⁺ (1) showing interactions between the BmpD protein and the ligand adenosine. Carbon atoms are shown as green, nitrogen as blue and oxygen as red spheres. Protein bonds are shown in dark green and ligand bonds in pink. Water molecules are shown as dark blue spheres and hydrophobic residues are shown as “eyelashes”. The green dashed lines show all potential hydrogen bonds between the protein and the ligand.

Reference

1. Laskowski R A, Swindells M B (2011). LigPlot+: multiple ligand-protein interaction diagrams for drug discovery. *J. Chem. Inf. Model.*, **51**, 2778-2786.
